# Retraction Note: LncRNA TUG1 was upregulated in osteoporosis and regulates the proliferation and apoptosis of osteoclasts

**DOI:** 10.1186/s13018-020-02000-1

**Published:** 2020-10-12

**Authors:** Ye Han, Chunying Liu, Ming Lei, Shaosong Sun, Wenkui Zheng, Yanan Niu, Xi Xia

**Affiliations:** 1 Department of Orthopaedics, Affiliated Hospital of Hebei University, Baoding City, 071000 Hebei Province People’s Republic of China; 2 Department of Pharmacology, School of Clinical Medicine, Hebei University, Baoding City, 071000 Hebei Province People’s Republic of China; 3 Department of Orthopaedics, Baoding First Central Hospital, No. 320, Great Wall North Street, Baoding City, 071000 Hebei Province People’s Republic of China

**Retraction Note: J Orthop Surg Res (2019) 14:416**

**https://doi.org/10.1186/s13018-019-1430-4**

The Editor-in-Chief has retracted this article [1] due to lack of evidence that the study has received ethics approval. After publication it has come to the Editor’s attention that in the body of the article the authors state that their study was approved by Baoding First Central Hospital. However, in the Ethics declarations section, the authors state that their study was approved by the Ethics Committee of the Maternity and Child Care Center of Liuzhou. The authors have not provided documentation of approval from an ethics committee for this study. The authors have not responded to any correspondence regarding this retraction.
